# Using oral care sets to reduce ventilator-associated pneumonia in intensive care units of tertiary care hospitals in a middle-income country: a multi-center study

**DOI:** 10.1017/ash.2025.84

**Published:** 2025-09-03

**Authors:** Akeau Emeritur

## Abstract

**Objectives:** A multi-center study aimed to determine the outcome of using oral care sets for cleaning the oral cavity of the patients admitted to the Intensive Care Units (ICU). **Methods:** Oral care sets which are single-use sets consisting of two toothbrushes with toothpaste, six 0.12% chlorhexidine swabs, and an oral moisturizer swab were developed. The ICUs of fourteen tertiary care hospitals participated in the study. All ICU nurses were asked to brush the patient’s teeth twice and clean the patient’s oral cavity with antiseptic six times daily. One thousand four hundred and two patients were recruited. The oral care sets were used with each patient on the first day they received mechanical ventilation until weaning. VAP surveillance was conducted to compare the VAP rate before and after the ICU used the oral care set. **Results:** Two hundred and sixty-six VAPs developed with an overall 34,731 ventilator days in the participating ICUs in 2022, before ICUs used oral care sets. The VAP rate was 7.66 per 1,000 ventilator days. The cost of antibiotic treatment was 5,134,621.74 Thai Bath. In 2023, after the ICUs used the oral care sets, 182 VAPs were developed. The overall ventilator day was 34,309. The VAP rate was reduced to 5.30 per 1,000 ventilator days. The cost of antibiotic treatment was reduced to 2,101,939.70 Thai Bath. One hundred and eighty-seven ICU nurses evaluated the benefit of the oral care set. Ninety-six-point eight percent of them agreed and strongly agreed that the single-use oral care set could prevent hospital- associated infections. Ninety-two-point five percent agreed and strongly agreed that only one nurse could clean the patient’s oral cavity, the oral care set helped reduce VAP occurrence (92%), the patient’s teeth and oral cavity were clean (92%), and ICU nurses could work conveniently (91.4%). **Conclusion:** The single-use oral care set can help reduce the VAP occurrence among patients admitted to the ICU.

